# Impact of generative AI in medical education in India: a systematic review

**DOI:** 10.3389/frai.2025.1704785

**Published:** 2025-12-17

**Authors:** Azfar Mateen, Visesh Kumar, Ajay Kumar Singh, Berendra Yadav, Mala Mahto, Atiq Hassan, Nazim Nasir

**Affiliations:** 1 Department of Forensic Medicine, Mahamaya Rajkiya Allopathic Medical College, Ambedkar Nagar, India; 2 Department of Biochemistry, Mahamaya Rajkiya Allopathic Medical College, Ambedkar Nagar, India; 3 Department of Physiology, Mahamaya Rajkiya Allopathic Medical College, Ambedkar Nagar, India; 4 Department of Biochemistry, All India Institute of Medical Science, Patna, India; 5 Department of Basic Medical Sciences, College of Applied Medical Sciences, King Khalid University, Abha, Saudi Arabia

**Keywords:** artificial intelligence ethics, ChatGPT, curriculum integration, generative AI, India, medical education, systematic review

## Abstract

**Background:**

The advent of generative Artificial Intelligence (AI) has presented a fundamental change in the approach to medical education across the world. In India, where the medical education is facing a shortage in faculties and resources, generative AI (GenAI) has the potential of transforming this. This systematic review summarizes the current evidence on the impact, student readiness, and various ethical challenges and barriers of integration of AI into the medical curriculum.

**Methods:**

We followed the Preferred Reporting Items for Systematic reviews and Meta-Analyses (PRISMA) guidelines and searched published articles in PubMed and Google Scholar from 2020 to 2025. The search yielded 19,777 articles, from which 11 studies focusing on Indian medical students were selected. The findings of these studies were analyzed using Laurillard’s six learning modes to gain a comprehensive pedagogical understanding.

**Result:**

Our study revealed a significant finding: while high awareness and positive perception towards AI have been shown by Indian medical students, most of the students lack formal training. These selected studies show that the students mostly use generative AI for clearing doubts, making assignments, and self-directed learning, shifting from the ‘Acquisition’ to ‘Inquiry’ and ‘Production’ modes of Laurillard’s learning. Comparative Analysis showed that GenAI tools outperform students on standard exams, thus showing their potential. However, certain challenges also exist, including the risk of misinformation, over-reliance, potential decrease in critical thinking, and ethical concerns of data privacy.

**Conclusion:**

Indian medical students are enthusiastically adopting GenAI, but their engagement is mostly unstructured and informal. A significant gap exists between the readiness of the students and the medical institutions. To maximize the potential use of GenAI, our institutions have to develop a structured curriculum, invest in faculty training, and establish ethical guidelines. Teamwork between policymakers, educators, and researchers is the need of the hour so that our future physicians will be ready to integrate AI-enabled healthcare.

**Syestematic review registration:**

https://doi.org/10.17605/OSF.IO/2MJVK.

## Introduction

Artificial Intelligence (AI), specifically generative AI (GenAI) models like ChatGPT, Gemini, and Claude, has added a new dimension to medical education in India, along with the rest of the world. One of the most important characteristics of GenAI is that it can generate its own text without manual input. GenAI can also simulate reasoning. GenAI is part of a larger ecosystem of Large Language Models (LLMs), which are increasingly used in healthcare education, diagnosis, and decision support (Jeyaraman et al., 2023).

Indian medical education is currently facing various challenges, like curriculum redundancy, the exponential increase in medical knowledge, and a decreasing faculty-student ratio. In view of these problems, GenAI has presented a promising solution by supporting content generation, self-directed learning, and simulated clinical reasoning (Barde et al., 2024; Mir et al., 2023).

In the rest of the world, AI tools have promoted personalized learning, clinical decision-making, and adaptive assessment (Sun et al., 2023; Rasouli et al., 2024). In many countries, AI applications have improved learning in diagnostics, radiology, teleconsultations, genomics, emergency preparedness, and patient communication through immersive simulations (Iqbal et al., 2021; Jung, 2022). Different AI-driven platforms are also helping the students to grasp complex biomedical concepts and address inconsistencies in traditional mentorship (Iqbal et al., 2021; Masters, 2019).

But, despite these advances, ethical and practical concerns that remain to be answered. It is common for GenAI to generate factually incorrect content, and it can also hallucinate, creating doubts about academic integrity (Jeyaraman et al., 2023; Sallam, 2023). In the Indian context, where there is already a disparity between digital literacy and infrastructure, the integration of GenAI with curricular alignment should be carefully planned, and there must be faculty training and ethical oversight for the same (Mir et al., 2023; Royal et al., 2014).

The readiness of medical students towards AI is complex. A cross-sectional study done in central India by the Medical Artificial Intelligence Readiness Scale for Medical Students (MAIRS-MS) scale indicates that overall readiness is moderate, but lots of variation is found in cognition domains (Dhurandhar et al., 2025). It shows that structured training programs of AI competencies are needed for both students and educators.

Curriculum development should occur in parallel. Indian medical educators have highlighted the need to update the MBBS curriculum and add new topics like genomics, digital health, and AI (Mir et al., 2023; Slavin and D'Eon, 2021). These curriculum reforms must be synchronized with global medical trends and prepare our doctors for AI-integrated clinical settings.

Against this background, this systematic review focuses on understanding the impact of generative AI on medical education in India. It also focuses on educational outcomes, learner readiness, ethical issues, and curriculum integration. The purpose of our study is to encourage the adoption of GenAI more responsibly and ethically by educators, researchers, and policymakers in India’s evolving medical education.

## Methods

### Protocol registration

A prospectively developed systematic review protocol was registered in the Open registries network (Open Science Framework, https://doi.org/10.17605/OSF.IO/2MJVK).

### Search strategy and selection criteria

This study followed the guidelines given by the Preferred Reporting Items for Systematic Reviews and Meta-Analyses (PRISMA) (Page et al., 2021). Our two authors performed detailed electronic search of publications using the PubMed database, which is one of the most widely used databases for medical research article and from Google Scholar, which is one of the most widely used search engine for research article. Our searches were limited to papers published in English from 2020 to 2025, as there has been an exponential increase in the use of AI in recent years. Search terms were structured to address medical education and GenAI.

#### PubMed (2577)

(“Generative Artificial Intelligence”[Mesh] OR generative artificial intelligence OR GenAI OR ChatGPT OR large language model* OR LLM*) AND (“Education, Medical”[Mesh] OR “Education, Medical, Undergraduate”[Mesh] OR “Education, Medical, Graduate”[Mesh] OR medical education OR health professions education OR graduate medical education OR undergraduate medical education OR clinical training) AND (impact OR effect OR influence OR outcome)

#### Google Scholar (17,200)

(“generative artificial intelligence” OR ChatGPT OR “large language model” OR GenAI OR LLM) AND (“medical education” OR “health professions education” OR “undergraduate medical education” OR “graduate medical education”) AND (impact OR effect OR outcome OR influence)

### Study inclusion and exclusion criteria

The inclusion criteria for this review were the following:

1. Studies published between 2020 and 2025.
2. Population -Indian medical students were considered.
3. Studies that show the impact of generative AI tools (like ChatGPT, Copilot, Gemini, etc.) on medical education.

Exclusion criteria include

1. Those studies in which non generative AI tools (like virtual reality or simulation studies were used).
2. Editorials, opinion articles, commentaries, letters, and blogs.
3. Articles containing only a theoretical framework.
4. Those studies in which no students were involved.

### Risk of bias assessment

The methodological quality of the selected studies was evaluated using the Joanna Briggs Institute (JBI) Critical Appraisal Checklist (Moola et al., 2020). Studies were appraised by three independent reviewers (AM, VK, and AKS), and any discrepancies were resolved by consensus.

### Analysis of the study

Using content analysis, we deductively coded the included papers and aligned them with Laurillard’s six learning modes (acquisition, inquiry, practice, production, discussion, and collaboration) (Laurillard, 1979).

## Results

### Study selection

**Figure 1:**
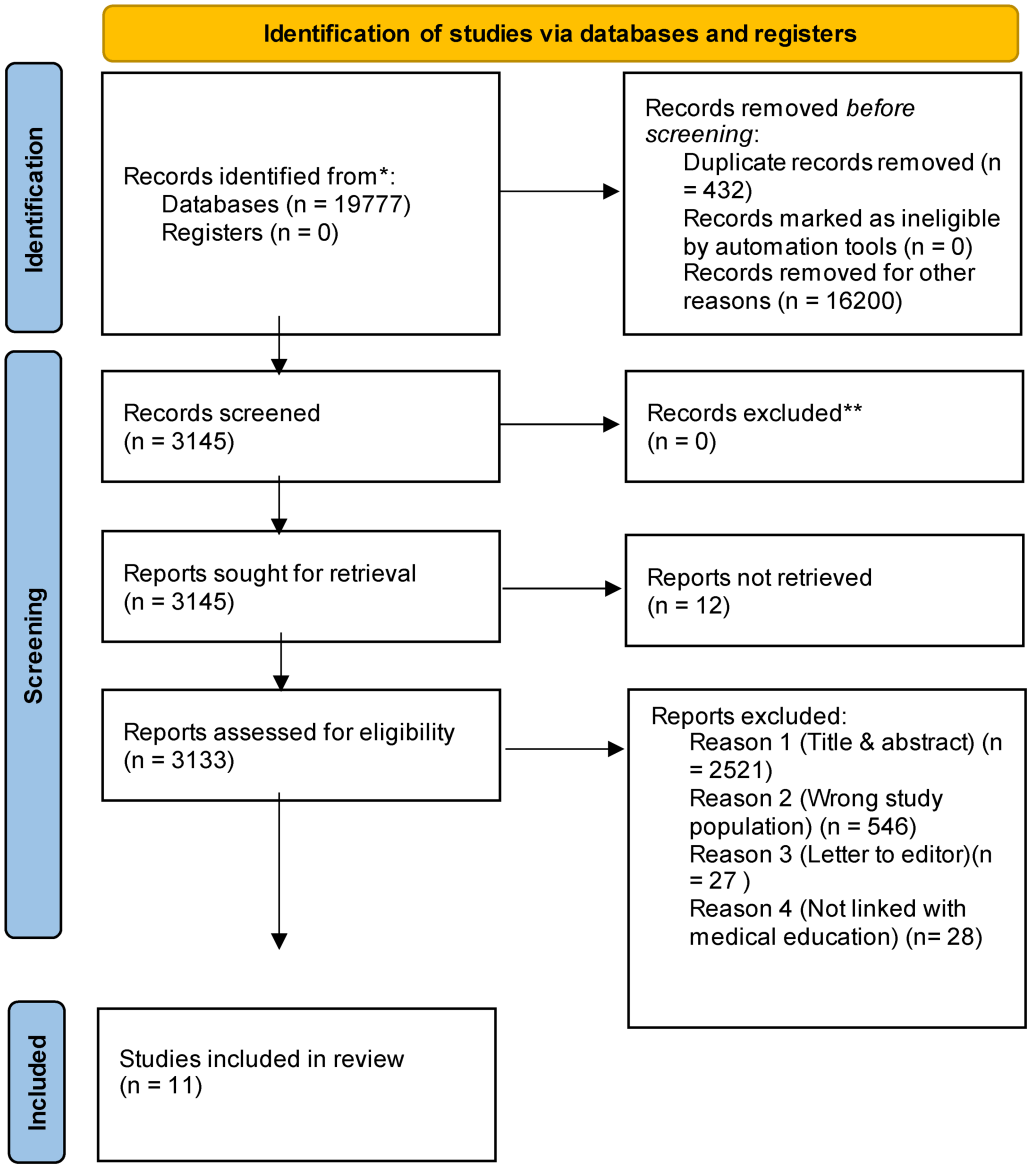
*From*: Page MJ, McKenzie JE, Bossuyt PM, Boutron I, Hoffmann TC, Mulrow CD, et al. The PRISMA 2020 statement: an updated guideline for reporting systematic reviews. BMJ 2021;372:n71. doi: https://doi.org/10.1136/bmj.n71 (Page et al., 2021). *Consider, if feasible to do so, reporting the number of records identified from each database or register searched (rather than the total number across all databases/registers). **If automation tools were used, indicate how many records were excluded by a human and how many were excluded by automation tools.

The electronic search retrieved 19,777 studies from the PubMed database and Google Scholar. After limiting the Google Scholar review to the first 1,000 results (as Google Scholar imposes a hard limit of 1,000 search results per query, 16,200 were not accessible). and removing 432 duplicates, 3,145 unique records were screened. Twelve articles were not accessible from our institution so, 3,133 reports assessed for eligibility. Screening titles and abstracts led to the exclusion of 2,521 records. The full texts of the remaining 612 reports were assessed for eligibility. Of these, 601 were excluded for not meeting the inclusion criteria, primarily due to the wrong study population, incorrect article type (e.g., letter to the editor), or a focus outside of medical education. This two-stage screening process resulted in the inclusion of 11 studies that met all eligibility requirements (Ahmad et al., 2024; Agarwal et al., 2025; Biri et al., 2023; Sorte et al., 2025; Sharma et al., 2025; Gandhi et al., 2024; Mondal, 2025; Ghosh et al., 2023; Rani et al., 2025; Mondal et al., 2025; Sharma et al., 2023). The detailed study selection process is illustrated in the PRISMA flow diagram (Figure 1).

Three reviewers (AM, VK, and AKS) performed the literature search independently to identify the studies for inclusion in the present review. AM, VK, AKS, and BY then extracted the pre-specified data from the studies. Any disagreements were resolved by discussion and consensus.

### Study and participant characteristics

For this systematic review, we analyzed 11 studies that evaluated the role of ChatGPT and other generative AI in relation to undergraduate medical education in India. Using qualitative interviews, cross-sectional surveys, and comparative performance analysis, the perceptions, attitudes, and experiences of students were explored. The characteristics of the included studies are summarized in Table 1.

**Table 1:** Study characteristics.

SNo	Author	Title	Journal	Year of publication	Study population	Method of data collection	Study design	Sample Size	Survey Area	Conclusion	Remark	ROB
1	Ahmad et al.	Exploring medical students’ outlook on the use of artificial intelligence in medical education: a multicentric cross-sectional study in Jharkhand, India	Cogent Education	2024	MBBS students, all phases	Online Google Form	Cross-sectional observational	417	Jharkhand, India	Widespread awareness of ChatGPT; students support integrating AI content with appropriate guidance and ethics safeguards.	Single center; online survey; convenience sampling.	8
2	Agarwal et al.	Evaluating the accuracy and reliability of large language models (ChatGPT, Claude, DeepSeek, Gemini, Grok, and Le Chat) in answering item-analyzed multiple-choice questions on blood physiology	Cures	2023	MBBS students at a medical college	Questionnaire-based survey	Cross-sectional	193	Rae Bareilly	Moderate knowledge and favorable attitudes toward AI/ChatGPT; students endorsed integrating AI training into the curriculum.	Single institution limits generalizability; self-reported outcomes.	9
3	Biri et al.	Assessing the utilization of large language models in medical education: insights from undergraduate medical students	Cures	2023	MBBS students	Google Form	Cross-sectional survey	370	Pan-India	Students are generally aware and interested; ethical concerns noted (per instrument focus).	Multicentric study	7
4	Sorte et al.	Future ready medicine: assessing the need for A.I. education in Indian undergraduate medical curriculum: a mixed-method survey of student perspectives	Journal of Education and Health Promotion	2025	Undergraduate medical students	Questionnaire survey	Cross-sectional, mixed-methods	219	Maharashtra, India	91% lacked prior AI training; strong consensus for integrating AI into IMU curriculum with experiential learning; no gender differences in interest.	Multi-department sample; still limited to select institutions.	7
5	Sharma et al.	Exploring undergraduate medical students’ perspectives towards artificial intelligence in healthcare: a qualitative study from India	JMA Journal	2024	Medical students	Semi-structured interviews/focus groups (per article type)	Qualitative study	124	India	Explores the lived experience of student use of ChatGPT; emphasizes guidance, ethics, and responsible use.	Single center	6
6	Gandhi et al.	Performance of ChatGPT on the India undergraduate community medicine examination: cross-sectional study	JMIR Formative Research	2024	Final year MBBS students’ exam papers (for comparison) and ChatGPT-3.5 outputs	Secondary analysis of internal assessment question papers; ChatGPT responses evaluated by independent assessors	Retrospective comparative evaluation (cross-sectional)	94 student scripts;	Hyderabad, India (publicly funded medical college)	ChatGPT scored 66.7% overall vs. students’ mean ~44%; responses were largely relevant/coherent/complete; suggested cautious, supervised integration.	Single institution; potential evaluator bias; contextual gaps for India noted by authors.	9
7	Mondal	Evolving resource use for self-directed learning in physiology among first-year medical students in a classroom setting	Illuminations	2025	First-year MBBS students	Open-ended question	cross-sectional	63	Jharkhand, India	AI tools, along with search engines, are the most commonly used resources.	Single-center, encouraging the students for the judicious use of both traditional and modern resources.	7
8	Ghosh et al.	Is ChatGPT’s knowledge and interpretative ability comparable to first professional MBBS (Bachelor of Medicine, Bachelor of Surgery) students of India in taking a medical biochemistry examination?	Cureus	2023	First-year MBBS students	Questionnaire immediately after classroom use of ChatGPT	Cross-sectional	101	India	AI (ChatGPT) outperformed almost all the students in both descriptive questions and MCQs	LLMs are getting better day by day, and soon they will start impacting medical education.	9
9	Rani et al.	Perception of medical students and faculty regarding the use of Artificial Intelligence (AI) in medical education: a cross-sectional study	Cureus	2024	MBBS students, Residents, and faculty	Online questionnaire	Cross-sectional	242 medical students	Jharkhand, India	Medical students acknowledged potential educational benefits and considered subjects that can be taught by integrating AI; they emphasized training, ethics, and policy frameworks.	There is an urgent need for changes in medical education and for the use of modern teaching methods to nurture AI competency and ethics.	8
10	Mondal et al.	A qualitative survey on the perception of medical students on the use of large language models for educational purposes	Advances in Physiology Education	2025 (first published online 2024-10-24)	Undergraduate medical students	Semi-structured, in-depth telephonic interviews; recorded, transcribed; thematic analysis (QDA Miner Lite v2.0.8)	Cross-sectional qualitative survey with convenience and snowball sampling;	25	Pan-India	Students perceived LLMs as useful for clarifying complex topics, note-making, MCQs, and assignments; concerns included errors, reliability, privacy; emphasized training and human oversight for responsible integration.	Three major themes were identified: usage scenario, augmented learning, and limitations/concerns. Most used ChatGPT-3.5.	5
11	Sharma et al.	Artificial Intelligence (AI) integration in medical education: a pan-India cross-sectional observation of acceptance and understanding among students	Scripta Medica (Brno)	2023	Medical students	Online questionnaire	Cross-sectional survey	730	India	To improve the current medical curriculum, there is a need to incorporate certain medical courses in order to create awareness of AI and newer technologies.	Nationwide survey; possible self-selection bias.	8

### Overall findings

Most studies (Sharma et al. (2023), Ahmad et al. (2024), Agarwal et al., 2025, Sorte et al. (2025), Ghosh et al. (2023), and Rani et al. (2025)) reported that medical students appreciated the potential benefits of various AI tools, particularly for simplifying complex concepts, clearing doubts, completing assignments, and practicing MCQs. Studies by Mondal (2025) and Mondal et al. (2025) provided deeper insights into the unsupervised use of LLM to improve self-directed learning and enhance students’ creativity and simulation-based learning.

A crucial aspect of this review is that it also includes objective measures of AI performance on an academic task. Comparative studies by Ghosh et al. (2023) and Gandhi et al. (2024) provided data comparing the marks obtained by LLMs with those of medical students (see Table 2).

**Table 2:** Studies providing Laurillard’s learning modes.

Laurillard’s learning mode	Number of papers addressing the mode	Papers that provide evidence (citations)
Acquisition	11	All 11 papers Sharma et al. (2023), Ghosh et al. (2023), Gandhi et al. (2024), Mondal (2025), Mondal et al. (2025), Agarwal et al. (2025), Ahmad et al. (2024), Biri et al. (2023), Sorte et al. (2025), Rani et al. (2025), and Sharma et al. (2025). *(All papers discuss how students gain knowledge about AI, whether formally or informally.)*
Inquiry	11	All 11 papers Sharma et al. (2023), Ghosh et al. (2023), Gandhi et al. (2024), Mondal (2025), Mondal et al. (2025), Agarwal et al. (2025), Ahmad et al. (2024), Biri et al. (2023), Sorte et al. (2025), and Rani et al. (2025); Sharma et al. (2024), *(All papers highlight the use of AI tools for asking questions, simplifying topics, and exploring concepts.)*
Practice	9	Sharma et al. (2023), Ghosh et al. (2023), Mondal (2025), Mondal et al. (2025), Agarwal et al. (2025), Ahmad et al. (2024), Biri et al. (2023), Sorte et al. (2025), and Rani et al. (2025). *(These papers discuss using AI for generating practice questions, simulations, and hands-on exercises.)*
Discussion	8	Sharma et al. (2023), Mondal (2025), Mondal et al. (2025), Ahmad et al. (2024), Biri et al. (2023), Sorte et al. (2025), Rani et al. (2025), and Sharma et al. (2024). *(These papers cover AI’s role in clarifying doubts, but also the concern that it might reduce human interaction.)*
Production	7	Ghosh et al. (2023), Gandhi et al. (2024), Mondal (2025), Mondal et al. (2025), Ahmad et al. (2024), Biri et al. (2023), and Sharma et al. (2024). *(These papers mention using AI to create assignments, presentations, and research proposals.)*
Collaboration	6	Mondal et al. (2025), Ahmad et al. (2024), Biri et al. (2023), Sorte et al. (2025), Rani et al. (2025), and Sharma et al. (2024). *(These papers highlight the student desire for interdisciplinary projects and the need for expert collaboration.)*

#### GenAI technology and Laurillard’s six learning modes

**Figure 2:**
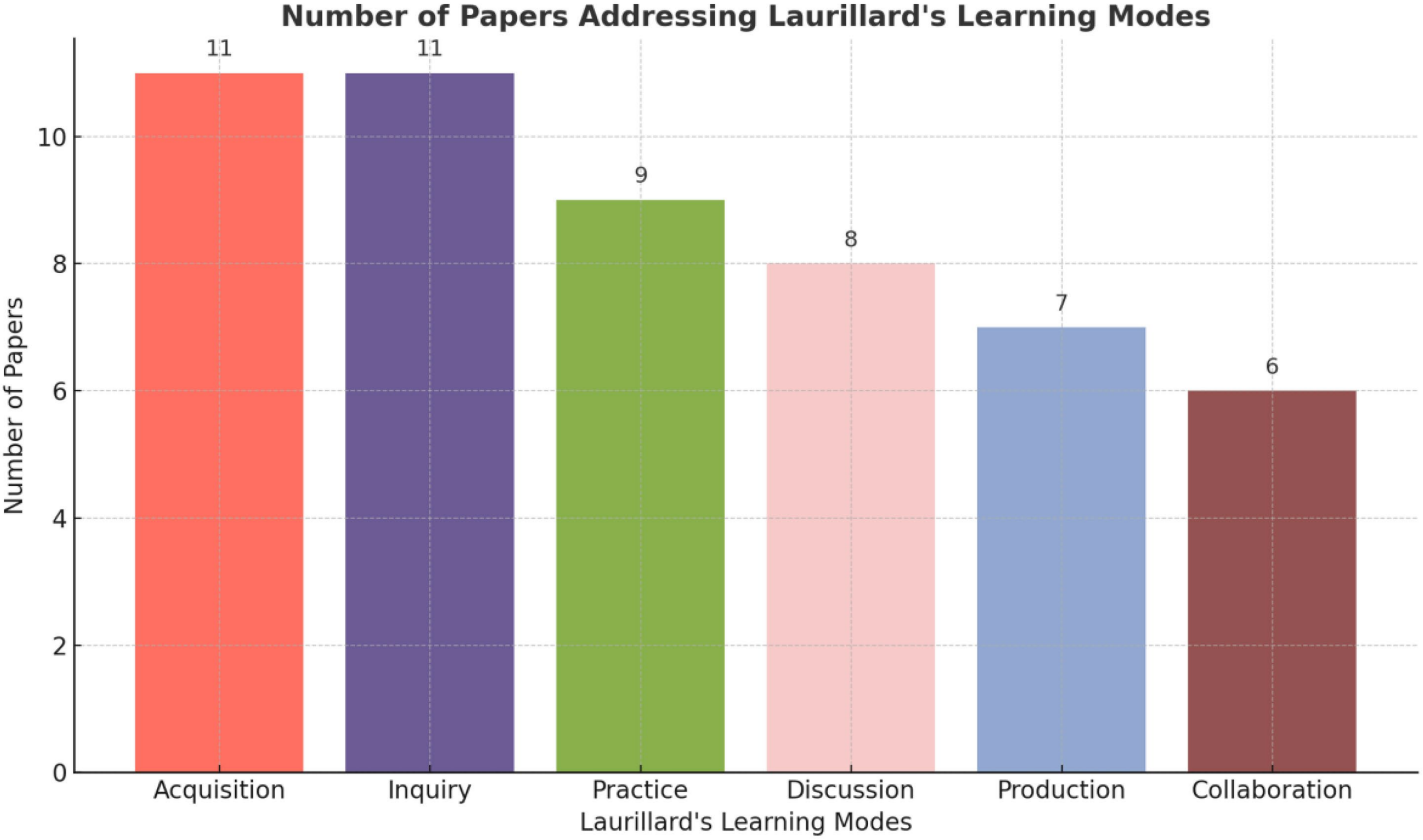
Number of studies addressing Laurillard’s learning modes.

A summary of the number of papers addressed to each learning mode is presented in Figure 2.

### Acquisition-learning through listening, reading, and watching

The acquisition theme has been reported in every study. Students primarily acquire AI knowledge through informal channels rather than formal instruction. According to Sharma et al. (2023), the primary sources of AI knowledge were media (55.8%), movies/TV series (34.2%), and peers (28.1%). The research by Ahmad et al. (2024) and Rani et al. (2025) both confirmed that the majority of learning occurs outside the formal curriculum by pointing out a discrepancy between students’ formal training in AI and their awareness of it.

### Inquiry-learning through investigation and exploration

Every study has shown that students are increasingly using AI tools (LLM chatbots) for inquiry-based learning in an unsupervised manner. Compared to a traditional textbook, Mondal (2025) found that the most popular resources for self-directed learning were search engines (61.9%) and LLM chatbots (60.32%). Ghosh et al. (2023) found that ChatGPT is an important inquiry tool. A Study by Sharma et al. (2023) found that students perceive AI as a valuable tool to improve diagnostic accuracy and to design customized treatment plans, rather than AI tools for patient treatment.

### Practice-learning through applying knowledge

In 9 out of 11 studies, the practice theme has been prominently highlighted. A Study by Sorte et al. (2025) found that 80% of students believed AI training should be experiential, with a preference for hands-on workshops (63.1%) and simulations (56.7%) showing the AI applications in clinical scenarios. The study by Rani et al. (2025) also found that students (88.62%) were interested in structured AI training, signaling a desire for conceptual understanding of AI tools along with the practical ability to use them responsibly and effectively.

### Production-learning through articulating and creating

This mode has been identified in 7 out of 11 studies. Students were using AI to complete their academic work more effectively. According to Mondal et al. (2025), students use LLMs in an unsupervised manner to create assignments, make presentations, and write more effectively. Ahmad et al. (2024) reported that students appraise AI as a tool for conducting research and similar activities, although the faculty supervision varied significantly. This classification will be contingent upon the LLM being used as a copilot for generating and articulating content rather than substituting student cognitive effort.

### Discussion-learning through conversation with peers and teachers

This mode was found in 8 out of 11 studies. According to Mondal et al. (2025), students turn to LLMs for immediate doubts clarification when the faculty members are not available, thus making the AI tool a substitute of faculty discussion. Sharma et al. (2023) found that a major concern of AI integration is its lack of empathy (43.7%). Our reason for considering this mode is to reflect the intention of the students which is to have a conversational style interaction for clarification of a concept thus substituting traditional discussion partner. We mainly focus on cognitive clarification rather than social or ethical reasoning development.

### Collaboration-learning through building on each other’s work

The Collaboration mode was identified in 6 out of 11 papers. Sorte et al. (2025) found that 53.5% of students wanted interdisciplinary teamwork between students from medical and other streams (e.g., Computer science or data science), highlighting that the effective AI integration needs both healthcare and technology understanding.

## Discussion

This systematic review presents a conundrum in the Indian medical education landscape because the students are enthusiastic about generative AI, but they lack a formal training and critical appraisal skill. The integration of AI into the medical curriculum is no longer just a future concept; it has become an immediate and complex challenge. Currently, there are three different levels of AI integration that have been revealed in our study: (1) Students using LLM tools without supervision and formal training. (2) Lack of AI fundamentals and a proper AI tool in the medical curriculum at present. (3) Minimal adoption of AI by faculty for developing educational materials.

Our study is unique because the findings have been analyzed through the lens of Laurillard’s conversational framework, suggesting that if a generative AI is to become a truly transformative educational partner, then the medical institutions should move beyond passive technology acquisition. Our study will explore the Laurillard’s six learning type so that a holistic roadmap can be taken for the integration of generative AI in Indian medical education. This study also reveals that the students are highly interested in learning AI but there exists a knowledge gap, so urgent, structured educational intervention are needed.

### Status of AI awareness and knowledge among Indian medical students

A consistent theme that emerged from our review is that among Indian medical students, there is a high level of awareness, but the formal knowledge level is low. For instance, Sharma et al. (2023) reported that 80.7% of 730 pan India respondent have heard about the AI whereas Rani et al. (2025) reported that 86.95% of 299 participants know how to use AI. This ‘awareness’ means basic functional knowledge of LLMs like ChatGPT rather than complex technical concepts like machine learning algorithms. However, 53.6% of students are having limited knowledge of AI application in the field of medicine as per the study conducted by Sharma et al. (2023). Similar findings were reported by Sharma et al. (2025), who found that the participant had varying knowledge about the advantages of using AI in healthcare. Sorte et al. (2025) found that 91.2% of students had never undergone formal AI training. These findings agreed with international findings in which Civaner et al. (2022) stated that 75.6% of the participants did not receive any training on AI in medicine. A Similar finding was also reported by Biri et al. (2023) in their Knowledge-Attitude framework in which it was found that although the students are showing interest in the integration of AI, but their knowledge was low. This finding is consistent with a systematic review by Abd-Alrazaq et al. (2023), in which the worldwide trend of having enthusiasm more than the actual competency in AI literacy among healthcare students was found.

### AI performance and educational efficacy

Perhaps most striking are the findings of comparative studies which examine the performance of AI in actual medical examinations against medical students and demonstrates the Large Language Models (LLMs) remarkable capabilities in medical education contexts. Ghosh et al. (2023) found that ChatGPT significantly outperformed medical students in a biochemistry examination, scoring 70% (140 marks out of 200), whereas the mean score of medical students was 96.4 ± 26.5. The evaluation of the medical examination by ChatGPT showed that ChatGPT passed the exam with an overall score of 66.7% indicating an adequate knowledge base as reported by Gandhi et al. (2024). This result shows that LLM can answer factual medical questions, but does not reflect whether the LLMs are capable of clinical reasoning and in patient care decisions or not. These results were similar to those of other international studies, such as Bharatha et al. (2024) and Meyer et al. (2024), which found that AI supersedes the students in medical assessment.

The superior performance of AI systems raises profound questions about current assessment methods and educational objectives. Agarwal et al. (2025) conducted a comparative analysis of six Large Language Models (LLMs) on blood physiology questions, revealing that Claude achieved 95% accuracy, DeepSeek 93%, and Grok 93%, significantly outperforming human students. This phenomenon challenges traditional notions of medical knowledge acquisition. It suggests the need for a fundamental reassessment of competency evaluation frameworks, as advocated by Mbakwe et al. (2023) in their critique of current medical education paradigms.

But in some of the studies like Wang and Liu (2023) found that ChatGPT lacks comprehension which human mind can do, also they found that certain contents were illogical, and it contains significant error. While Wójcik et al. (2024) reported that ChatGPT has only 55.8% correct response rate to the questions given which is contradictory to the findings of high correct response rate found in the studies of our review. These inconsistencies show the importance of why students must know how to critically assess the response generated by LLM rather than accepting it blindly.

## Student perceptions and acceptance

Across the studies, it reveals that the perception of medical students is very positive towards integrating AI into their curriculum. Sharma et al. (2023) found that 80.7% of study participants knew how to use AI and 46.8% supported that AI should be integrated in medical curriculum. Sorte et al. (2025) also reported that 84.3% of students are interested in the integration of AI in the undergraduate medical curriculum. Sharma et al. (2025) also found that the participants (57.7%) believed that the AI should be integrated into medical education for better preparation for future challenges. Similar findings were found in the study of Ahmad et al. (2024), in which it was reported that 85.7% of male and 80% of female participants were interested in the integration of AI in medical curriculum and assessment. This positive attitude towards AI shows that the students are keen to adapt to digital learning.

Three major themes in student perception were identified by Mondal et al. (2025): Usage scenarios, Augmented learning, and Limitations. The most commonly used resources for self-directed learning (SDL) as reported by Mondal (2025) was search engine (61.9%) and LLM chatbots (60.32%) whereas only 26.98% of the participants uses traditional textbooks for learning. This transition shows that unsupervised use of LLM by the students for inquiry driven learning persist despite having no formal training. This fundamental change in approach towards learning shows a pedagogical shift from Laurillard’s ‘learning through acquisition’ (from textbook) to ‘learning through inquiry’ (interaction with chatbots), which provides a strong justification for formal curriculum integration of AI so that the students learn appropriate use of LLM instead of unsupervised adaptation.

### Educational applications and learning enhancement

The findings identify a few areas for AI integration, including healthcare, medical image analysis, and the development of machine learning algorithms for curriculum development. Biri et al. (2023) identified that there are multiple ways in which students use LLM tools in unsupervised contexts like simplifying complex concepts, making assignments and summarizing the long topics, these activities are aligned with the ‘Learning through production’ where students express their understanding by creating an artifact.

The diagnostic capabilities of AI tools are also promising for medical education. Participants in Sorte et al. (2025) found that there is a potential in AIs to enhance the diagnostic accuracy (73.3%), in facilitating data analysis (71.9%), and also in improving the treatment plan (66.4%) suggesting how AI will be used in future clinical practice. Similar findings were also reported by Rani et al. (2025) in which the majority of participants reported that by integrating AI systems, we could improve particular areas of medical education, like diagnosis (154, or 51.5%), clinical reasoning (51, or 17.1%), radiology (50, or 16.7%), pathology slides (31, or 10.4%). This suggests that the students need a specialty AI application in their curriculum and the use of AI in a supervised, simulated clinical scenario. These preferred learning methods are aligned with Laurillard’s ‘Learning through practice’.

Hands-on learning experience is the most preferred method of learning among participants across the studies. Sorte et al. (2025) found that the preferred learning methods of AI were practical training with AI tools (80%), workshops (63.1%) and simulations (56.7%) respectively whereas in Sharma et al. (2023) the preferred learning methods for AI were workshops (45.2%), lectures (31.1%), online resources (33.7%) and extracurricular activities (32.1%) respectively. The clear preference for practical, hands-on application suggests that the medical education should move beyond passive technology acquisition and instead focus on learning through practice, production, and collaboration to incorporate AI competency.

### Challenges and barriers to implementation

Overall, the attitude of students is positive towards AI, but few studies have highlighted the barriers to its implication due to a lack of formal training and the absence of trained faculty in AI concepts and tools. Major concerns were regarding formal training. Sorte et al. (2025) found that 91.2% of the participants have not taken AI training in education. Gandhi et al. (2024) evaluated that the ChatGPT 3.5 is not capable of generating images highlighting a major limitation of its use in subjects like pathology and radiology where teaching is visual dependent.

Certain challenges were also reported by global studies. For instance, the study by Bagde et al. (2023) reported that the accuracy of AI while answering fluctuates from 18.3 to 100%. This vast range removes the notion of uniform reliability. Rabbani et al. (2025) reported that the generative AI particularly LLM’s tend to hallucinate, that is it has a habit of generating information, non-existing data and references which looks original. These limitations are mainly problematic when AI is used without supervision, as students may not be able to identify AI-generated errors. Equally concerning is the chance of intellectual dependency and cognitive atrophy (Gerlich, 2025). Medical education is not only about the ability to retrieve information, instead the student should inculcate the habit of clinical reasoning and should face the complexity and uncertainty with resilience (Ilgen et al., 2019; Ng et al., 2025; McMahon et al., 2025). When students depend too much on AI tools to interpret and simplify learning material, they may appear knowledgeable without involving the cognitive domain needed for understanding (Nadim and Di Fuccio, 2025).

Biri et al. (2023) reported that the students could not critically evaluate AI-generated output without basic training in AI. Similar findings were also reported by Safranek et al. (2023), in which it was suggested that to bridge the gap between awareness and effective utilization of AI, a formal practical training on LLM’s application is required.

Sharma et al. (2023) also reported a few challenges of introducing AI in medical education. Among the participants, 56% cited limited resources as the biggest hurdle, and almost half (45.8%) showed concerns about faculty members’ expertise, which is necessary to teach AI effectively. Sharma et al. (2023) also reported a few challenges of introducing AI in medical education, over half of the participants (56%) cited limited resources as the biggest hurdle.

### Ethical considerations and concerns

The ethical dimensions of AI integration received significant attention in most of the studies. Medical students expressed a major concern about over-reliance on AI. Participants of Sharma et al. (2023) highlighted certain drawbacks to AI integration that were, overreliance on AI (49.2%) followed by a perceived lack of empathy (43.7%) and concerns about patient privacy (37%) respectively. This points out the limitation of current technology in providing ‘Learning through discussion’ as currently AI tools cannot replicate the nuances of peer-to-peer social learning. Similar findings were reported by Ahmad et al. (2024), who found that respondents expressed fear of over-reliance in AI and were also concerned about the job security and data protection.

These findings were aligned with various global studies. Participants in this study showed concern about using AI tools in relation to data protection and patient privacy, these concerns were also highlighted by Fazakarley et al. (2023). Medical students were also concerned about various ethical issues related to academic honesty, data protection, plagiarism, and the lack of cultural awareness in AI systems as reported by Salih (2024). Weidener and Fischer (2023) reported that ethical considerations, privacy issues, and regulatory frameworks should be integrated into the use of AI in medical training. The research by Chan and Zary (2019) also found the ethical dilemmas of incorporating AI into medical education, especially in teaching empathy.

For medical education, ethical guidelines on AI in healthcare by ICMR (2023) imply embedding AI ethics in the medical curriculum, strengthening faculty in AI, training to validate AI tools, recognizing bias, and maintaining explainability in clinical use.

### Curricular integration and future directions

These studies give valuable insight into the development of AI integration in the medical education curriculum. The key areas identified by Sorte et al. (2025) for AI integration in medical education are AI in healthcare (62.2%), machine learning algorithms (59.4%), and AI algorithms for medical image analysis (70.5%). Students (52.5%) in this study also wanted collaboration between medical and computer science students, suggesting that an interdisciplinary integrated learning approach should be considered. This aligns with Laurillard’s ‘Learning through collaboration’.

A study by Chouvarda et al. (2019) highlighted the importance of interdisciplinary collaboration among healthcare professionals, computer scientists, and engineers. It also reports that effective health technologies can be developed only when various professionals from different fields work as a team. Similar findings were also reported by Breil et al. (2010), whose study participants were medical and informatics students, who showed that interdisciplinary and integrated learning methods were beneficial for medical students.

### Limitations and research gap

Our analysis has a few limitations. First, only a few studies were multicentric, making it difficult to generalize the findings to the whole Indian medical education system, which is highly diverse. Second, most of the data were collected through self-administered questionnaires, which may introduce response bias. Another major gap is that till date no longitudinal studies have been available, which can tell the long-term impact of AI tools use on learning outcomes and clinical competency.

Furthermore, the studies primarily focused on student perceptions rather than actual learning outcomes or clinical performance impacts. More research on medical academicians’ knowledge, attitudes, and perceptions is needed, as academicians are pillars of medical education, and the integration of AI into medical education requires their perception.

## Conclusion

An extensive review of the 11 studies included in our study reveals that the Indian medical students are eager to integrate AI tools in the medical curriculum, but the infrastructure of the institutions cannot keep up with this demand. A successful integration is only possible after addressing the current knowledge gap through structured curriculum development, along with capacity building of the faculty. It is high time for Indian medical institutions to integrate AI tools into their curricula, but this can only happen through immediate coordinated action among different stakeholders, policymakers, educators, and researchers. This coordinated action may be guided by two core imperatives based on evidence:

1. Moving beyond passive technology acquisition, as students are already using AI, medical institutions should shift their curriculum towards experiential learning and Laurillard’s higher modes—Practice, Production, and Collaboration.
2. Reassessment of competency so that in future faculty should be able to critically appraise AI output in the assessment.

But the rapid pace of AI technological advancement means that findings may become obsolete quickly, necessitating continuous monitoring and reassessment.

## Data Availability

The original contributions presented in the study are included in the article/supplementary material, further inquiries can be directed to the corresponding author.
